# The utility of 16S rRNA gene sequencing on intraoperative specimens from intracranial infections: an 8-year study in a regional UK neurosurgical unit

**DOI:** 10.1080/02688697.2021.2016620

**Published:** 2021-12-20

**Authors:** Timothy D. Shaw, Tanya Curran, Stephen Cooke, Ronan McMullan, Michael Hunter

**Affiliations:** a Wellcome-Wolfson Institute for Experimental Medicine, Queen’s University Belfast, Belfast, UK; b Department of Medical Microbiology, Belfast Health and Social Care Trust, Belfast, UK; c Regional Neurosciences Unit, Belfast Health and Social Care Trust, Belfast, UK; d Infectious Diseases Unit, Belfast Health and Social Care Trust, Belfast, UK

**Keywords:** 16S rRNA gene sequencing, intracranial infection, microbiological diagnosis, antimicrobial choice

## Abstract

**Background:**

Optimal management of intracranial infections relies on microbiological diagnosis and antimicrobial choice, but conventional culture-based testing is limited by pathogen viability and pre-sampling antimicrobial exposure. Broad-range 16S rRNA gene sequencing has been reported in the management of culture-negative infections but its utility in intracranial infection is not well-described. We studied the efficacy of 16S rRNA gene sequencing to inform microbiological diagnosis and antimicrobial choice in intracranial infections.

**Methods:**

This was a retrospective study of all intraoperative neurosurgical specimens sent for 16S rRNA gene sequencing over an 8-year period at a regional neurosurgical centre in the UK. Specimen selection was performed using multidisciplinary approach, combining neurosurgical and infection specialist discussion.

**Results:**

Twenty-five intraoperative specimens taken during neurosurgery from 24 patients were included in the study period. The most common reason for referral was pre-sampling antimicrobial exposure (68%). Bacterial rDNA was detected in 60% of specimens. 16S rRNA gene sequencing contributed to microbiological diagnosis in 15 patients and informed antimicrobial management in 10 of 24 patients with intracranial infection. These included targeted antibiotics after detection of a clinically-significant pathogen that had not been identified through other microbiological testing (3 cases), detection of commensal organisms in neurosurgical infection which justified continued broad cover (2 cases) and negative results from intracranial lesions with low clinical suspicion of bacterial infection which justified avoidance or cessation of antibiotics (5 cases).

**Conclusion:**

Overall, 16S rRNA gene sequencing represented an incremental improvement in diagnostic testing and was most appropriately used to complement, rather than replace, conventional culture-based testing for intracranial infection.

## Background

The prognosis of brain abscess has improved substantially over the last 50 years, due to advances in imaging, minimally-invasive neurosurgical procedures and protocoled antimicrobial therapy.^1^ However, microbiological diagnosis and antimicrobial rationalisation continues to rely on culture and susceptibility testing of intra-operative specimens. These specimens are culture-negative in around a third of cases, often due to the use of pre-operative antibiotics or presence of fastidious organisms.^2^ The dependence on prolonged, empirical antibiotic therapy in intracranial infections is well-recognised, as are the accompanying risks of antimicrobial resistance and drug toxicity.^3^^,^^4^ Therefore, there is a need to develop rapid and accurate diagnostic testing of intracranial specimens to inform antimicrobial choices and improve patient outcomes.

Molecular diagnostics encompass a range of nucleic-acid based laboratory techniques that can retrieve microbiological information from genomic analysis.^5^ Broad-range 16S rRNA gene sequencing can detect bacteria by amplifying a region of bacterial ribosomal RNA that is conserved at species-level, which is then sequenced and matched to a database of known species-specific sequences.^6^ It is a relatively new molecular diagnostic test which, though not routinely performed at many clinical laboratories, is available at some reference centres. The primary advantage of this technique is that it requires only genetic material for bacterial identification, compared to culture-based methods that require viable organisms.

The use of broad-range 16S rRNA gene sequencing to determine management of culture-negative infections has been described in small retrospective studies, some of which have included cases of intracranial infection.^7^^,^^8^ However, differences in the specimen selection process and availability of 16S rRNA gene sequencing between jurisdictions may affect its utility in intracranial infections. The clinical scenarios in which 16S rRNA gene sequencing on intraoperative neurosurgical specimens is most useful remain unknown.

We studied the utility of a multi-disciplinary approach at our centre in selecting intraoperative specimens for 16S rRNA gene sequencing over an 8-year period. Our aims were to determine the efficacy of 16S rRNA gene sequencing to inform microbiological diagnosis and antimicrobial choice in intracranial infections.

## Methods

### Setting

The Northern Ireland Neurosurgical Unit is based in the Belfast Health and Social Care Trust. It provides a regional adult and paediatric neurosurgical service for the population of Northern Ireland (approximately 1.8 million people). It is a 36 bedded unit that manages 10–15 cases of spontaneous intracranial infections and 30–40 cases of neurosurgical site infections per year. The Trust has had access to broad-range 16S rRNA gene sequencing through the Bacteriology Reference Department at Public Health England since 2012. The typical turn-around time from date of sending specimen to receiving result is 7 days. 16S rRNA gene sequencing is not a routine test for intracranial specimens and requests are assessed by an infection specialist (clinical microbiologist or infectious diseases physician) prior to sending. Specimens from neurosurgical cases are identified for 16S rRNA gene sequencing via a number of multi-disciplinary interactions. These include (i) a weekly joint ward round with consultant neurosurgeons and clinical microbiologists (ii) an inpatient referral pathway from neurosurgery to infectious diseases for Outpatient Parenteral Antibiotic Therapy and (iii) a weekly multidisciplinary meeting of infection specialists (clinical microbiologists, adult infectious diseases physicians and paediatric infectious diseases physicians). Specimens must be identified for 16S rRNA gene sequencing before they are discarded after prolonged culture (typically 14 days from time of processing).

### Data acquisition and analysis

A retrospective review was undertaken of all intraoperative neurosurgical specimens from patients sent for 16S rRNA gene sequencing analysis from January 2013 until January 2021. These were correlated with the patient’s laboratory, electronic and paper records. Data was collected on patient sex, age, specimen type, clinical diagnosis and site of infection, exposure to pre-operative antimicrobials, culture result, 16S rRNA gene sequencing result and antimicrobial course. A chart review was conducted to ascertain the role of 16S rRNA gene sequencing in clinician decisions over microbiological diagnosis and antimicrobial choice.

## Results

### Study population

Twenty-five specimens from 24 patients were included during the 8-year study period (Table 1). One patient had two specimens sent 8 weeks apart following neurosurgical procedures. The majority of cases from whom specimens were sent were male (15/24; 63%). The median age was 44 (IQR 31–53). Most patients had a clinical diagnosis of community-acquired intracranial infection (15/24, 63%), followed by healthcare-associated intracranial infection (5/24; 25%) and non-infective conditions (4/24; 17%).

**Table 1.** Results of 16S rRNA gene sequencing on intracranial specimens and contribution to patient management.

Sex	Age bracket	Clinical diagnosis	Neurosurgi site	Pre-sampling antibiotic exposure	Type of specimen	Culture result	Other relevant results	Indication for 16S rRNA gene sequencing	16S rRNA gene sequencing result	Contribution to microbiological diagnosis	Contribution to antimicrobial choice	Antibiotic choice
Community-acquired intracranial infection												
F	<18	Subdural empyema	Right temporal lobe	Yes	Fluid - subdural	NG	Histology: dural inflammation	Pre-sampling antimicrobial exposure	16S rDNA not detected	Negative with no contribution to diagnosis	No	Ceftriaxone
M	<18	Subdural empyema	Left temporal lobe	Yes	Pus - brain abscess	Streptococcus intermedius (on enrichment)	Blood culture isolated Streptococcus intermedius	Pre-sampling antimicrobial exposure	99% sequence homology to S. intermedius, S. anginosus and S. constellatus	Positive in concurrence with other microbiological tests	No	Ceftriaxone + Metronidazole ↓ Co-amoxiclav
M	<18	Subdural empyema	Right frontal lobe	Yes	Pus - brain abscess (craniotomy)	Streptococcus intermedius	Blood culture isolated Streptococcus intermedius	Pre-sampling antimicrobial exposure	99% sequence homology to S. intermedius, S. anginosus and S. constellatus	Positive in concurrence with other microbiological tests	No	Ceftriaxone
F	<18	Subdural empyema	Frontal lobe	Yes	Fluid - frontal subdural	NG	Blood culture isolated Streptococcus pneumoniae	Pre-sampling antimicrobial exposure	16S rDNA not detected	Negative with no contribution to diagnosis	No	Cefotaxime
M	18–29	Intracranial abscess	Frontal lobe	Yes	Pus - frontal abscess (craniotomy)	NG	**Nil**	Pre-sampling antimicrobial exposure	16S rDNA not detected	Negative with no contribution to diagnosis	No	Meropenem + vancomycin + metronidazole
F	30–49	Intracranial abscess	Right occipital lobe	No	Pus - occipital abscess (craniotomy)	Corynebacterium sp., Micrococcus luteus, Kytococcus sedantarius (all on enrichment)	Nil	Culture negative specimen without pre-sampling antimicrobial exposure	Prevotella sp., Moraxella sp. and Fusobacterium nucleatum	Positive with definitive result in absence of other positive tests	Yes - narrow spectrum	Ertapenem + metronidazole ↓ Amoxicillin
F	30–49	Intracranial abscess	Frontal lobe	No	Pus - frontal abscess (aspirate)	NG	None	Culture negative specimen without pre-sampling antimicrobial exposure	99% sequence homology to S. intermedius, S. anginosus and S. constellatus	Positive with definitive result in absence of other positive tests	Yes - narrow spectrum	Vancomycin + meropenem ↓ Meropenem
F	30–49	Subdural empyema	Left frontal lobe	Yes	Pus - frontal subdural (craniotomy)	NG	None	Pre-sampling antimicrobial exposure	99% sequence homology to S. intermedius, S. anginosus and S. constellatus	Positive in concurrence with other microbiological tests	No	Ceftriaxone + Metronidazole
F	30–49	CNS toxoplasmosis	Multiple ring-enhancing lesions	Yes	Tissue - brain	NG	Histology: non-specific inflammation and oedema; immunohisto chemistry negative for microorganisms	Pre-sampling antimicrobial exposure	16S rDNA not detected	Negative in concurrence with exclusion of bacterial infection	Yes - narrow spectrum	Ceftriaxone + amoxicillin ↓ Sulfadiazine
M	30–49	Disseminated intracranial abscesses	Multiple	Yes	Pus - frontal abscess (craniotomy)	NG	Histology: inflammation and neovascularisation	Pre-sampling antimicrobial exposure	99% sequence homology to S. intermedius, S. anginosus and S. constellatus	Positive with definitive result in absence of other positive tests	No	Ceftriaxone + Metronidazole
M	30–49	Intracranial abscess	Left frontal lobe	No	Tissue - brain abscess	Not done	Histology: inflammatory reaction representative of abscess	Specimen not processed correctly for culture	16S rDNA not detected	Negative with no contribution to diagnosis	No	Ceftriaxone + Metronidazole
M	50–69	Disseminated intracranial abscesses	Multiple	Yes	Tissue - abscess (craniotomy)	Nocardia farcinica	Histology: staining positive for nocardia-like microorganisms	Pre-sampling antimicrobial exposure	Nocardia farcinica	Positive in concurrence with other microbiological tests	No	Imipenem ↓ Ceftriaxone + ciprofloxacin
					Tissue - brain abscess (craniotomy)	Enterococcus faecium (significance unclear)	Histology: staining positive for nocardia-like microorganisms	Pre-sampling antimicrobial exposure	Nocardia farcinica	Positive in concurrence with other microbiological tests	No	Ceftriaxone + ciprofloxacin ↓ Ciprofloxacin
M	50–69	Disseminated intracranial abscesses	Multiple	Yes	Pus - brain abscess	Fusobacterium nucleatum	None	Pre-sampling antimicrobial exposure	Fusobacterium sp.	Positive in concurrence with other microbiological tests	Yes - narrow spectrum	Meropenem + Metronidazole ↓ Clindamycin + Co-trimoxazole
M	50–69	Subdural empyema	Left parietal lobe	Yes	Tissue - subdural membrane (craniotomy(	Fusobacterium sp.	None	Pre-sampling antimicrobial exposure	Fusobacterium sp.	Positive in concurrence with other microbiological tests	No	Ceftriaxone + Metronidazole ↓ Meropenem + Metronidazole
M	50–69	Pott's puffy tumour with skull osteomyelitis	Frontal bone	Yes	Pus - forehead	Mixed anaerobes (on enrichment)	Histology: chronic inflammatory change	Pre-sampling antimicrobial exposure	Prevotella oris and Porphyromonas endodontalis	Positive with definitive result in absence of other positive tests	Yes - narrow spectrum	Ceftriaxone + Metronidazole ↓ Co-amoxiclav + Metronidazole
Healthcare-associated intracranial infection												
F	30–49	Post-operative skull osteomyelitis	Occipital skull bone flap	Yes	Tissue - cranial wound	Corynebacterium striatum (on enrichment)	Wound swabs isolated Staphylococcus aureus and mixed anaerobes	Pre-sampling antimicrobial exposure	Corynebacterium striatum	Positive in concurrence with other microbiological tests	No	Teicoplanin + metronidazole ↓ Linezolid and metronidazole ↓ Clarithromycin + metronidazole
M	30–49	Post-operative extradural empyema	Right frontal lobe	Yes	Pus - intracranial (craniotomy)	NG	None	Pre-sampling antimicrobial exposure	Propionibacterium acnes	Positive but significance unclear	No	Flucloxacillin + ceftriaxone + metronidazole
F	50–69	Post-operative extradural empyema	Left temporal lobe	Yes	Tissue - dura	NG	Histology: bone osteonecrosis with acute inflammation	Pre-sampling antimicrobial exposure	Propionibacterium acnes	Positive but significance unclear	Yes - appropriate broader spectrum	Vancomycin + ceftriaxone + metronidazole
M	>70	Post-operative subdural empyema	Right parietal lobe	Yes	Pus - subdural	NG	None	Pre-sampling antimicrobial exposure	Propionibacterium acnes	Positive but significance unclear	No	Meropenem
Non-infective intracranial condition												
M	18–29	CNS degenerative disorder of unknown aetiology	Right frontal lobe	Yes	Tissue - brain biopsy	Staphylococcus epidermidis (on enrichment)	Histology: non-specific chronic reactive inflammation	Intracranial lesion of unknown aetiology	16S rDNA not detected	Negative with no contribution to diagnosis	No	Vancomycin + ciprofloxacin
M	30–49	CNS lymphoma	Left parietal lobe	No	Tissue - brain biopsy	NG	Histology: diffuse B-cell lymphoma	Intracranial lesion of unknown aetiology	16S rDNA not detected	Negative in concurrence with exclusion of bacterial infection	Yes - avoidance of antibiotics	None
M	30–49	CNS mitochondrial disorder	Multiple	No	Tissue - cortex	NG	Histology: non-specific meningoencephalitis with negative microbiological stains	Intracranial lesion of unknown aetiology	16S rDNA not detected	Negative in concurrence with exclusion of bacterial infection	Yes - avoidance of antibiotics	None
F	50–69	Post-operative non-infective arachnoiditis	Right frontal lobe	Yes	Tissue - brain cortex; dura (craniotomy)	NG	Histology: chronic inflammatory change; immunohisto chemistry negative for microorganisms	Intracranial lesion of unknown aetiology	16S rDNA not detected	Negative in concurrence with exclusion of bacterial infection	Yes - avoidance of antibiotics	None
M	>70	CNS autoimmune disorder	Right fronto-parietal lobe	No	Tissue - dura	NG	Histology: non-specific inflammation	Intracranial lesion of unknown aetiology	16S rDNA not detected	Negative in concurrence with exclusion of bacterial infection	Yes - avoidance of antibiotics	None

NG: no growth; CNS: central nervous system.

### Rationale for 16S rRNA gene sequencing

Three-quarters of cases (18/24) had received antimicrobial therapy prior to neurosurgical sampling and this was the most common reason for requesting 16S rRNA gene sequencing (17/24, 68%). In two cases (8%), specimens were requested for 16S rRNA gene sequencing from culture-negative abscess without prior antibiotic exposure. There were four cases (17%) in which 16S rRNA gene sequencing was sent during work-up for an intracranial lesion of unknown aetiology with low clinical suspicion of infection.

### Effect of 16S rRNA sequencing results on microbiological diagnosis

Bacterial rDNA was detected in 15 of 25 specimens (60%). Eight cases had 16S rRNA gene sequencing reports that concurred with the results of other microbiological testing (including blood culture, CSF or enrichment culture of intracranial specimens). In these instances, microbiological diagnosis had been determined by other tests and 16S rRNA gene sequencing results did not influence antimicrobial choice. In one case, *Nocardia farcinica* was both isolated from intracranial specimen on culture and detected on 16S rRNA gene sequencing. However, when neurosurgical sampling was repeated on appropriate antimicrobial cover 8 weeks later, the specimen was culture negative but still positive for *Nocardia* on 16S rRNA gene sequencing.

Seven patients had positive 16S rRNA gene sequencing results in the setting of culture-negative infection. These included two cases with *Streptococcus milleri* group, two cases with multiple fastidious organisms (including *Prevotella* sp., *Moraxella* sp., *Fusobacterium nucleatum* and/or *Porphyromonas* endodontalis) and three cases with commensal organisms (*Propionibacterium acnes*) detected.

### Effect of 16S rRNA sequencing results on antimicrobial management

Antimicrobials were rationalised in three of four cases in which clinically-relevant bacteria were detected by 16S rRNA gene sequencing in the absence of other positive tests. These were:

- Ertapenem and metronidazole rationalized to oral amoxicillin for occipital lobe abscess with in which 16S rRNA gene sequencing detected *Prevotella* sp., *Moraxella* sp. and *Fusobacterium nucleatum*
- Vancomycin and meropenem rationalised to meropenem only for community-acquired frontal lobe abscess with severe sepsis in which 16S rRNA gene sequencing detected *Streptococcus intermedius.* Although further rationalisation to a narrower beta-lactam agent was discussed, the responsible clinician decided to complete the course with meropenem.
- Ceftriaxone and metronidazole rationalized to co-amoxiclav and metronidazole for frontal bone osteomyelitis (Pott’s Puffy tumour) in which 16S rRNA gene sequencing detected *Prevotella oris* and *Porphyromonas endodontalis.*

In three cases, 16S rDNA of commensal organisms was detected (*Propionibacterium acnes* from post-operative intracranial infection in all three instances) and the clinical significance of this was unclear. In two of these cases, this result was used to justify continued broader antimicrobial cover.

There were 10 specimens in which no 16S rDNA was detected. These results contributed to the exclusion of a bacterial infection for five patients (in combination with other findings) and was used to justify cessation or avoidance of antimicrobial therapy. One of these patients was rationalised from empirical antibiotics to targeted therapy for CNS toxoplasmosis based on negative 16S rRNA gene result, HIV infection and neuroradiological findings. The other four of these cases had specimens sent on intracranial lesions with low clinical suspicion of infection.

## Discussion

In this study of 24 patients, 16S rRNA gene sequencing on intraoperative neurosurgical specimens contributed microbiological diagnosis in 15 cases and antimicrobial management in 10 cases. We identified three clinical scenarios in which 16S rRNA gene sequencing results shaped clinician decisions at our centre:

- Detection of clinically significant organisms in the setting of community-acquired intracranial infection, leading to rationalisation of broad-spectrum empirical antimicrobial regimens
- Detection of commensal organisms in the setting of post-neurosurgical infection, justifying continued broad-spectrum cover
- Negative results in the setting of low clinical suspicion of bacterial infection, justifying the avoidance or cessation of antimicrobials

Antimicrobial stewardship based on microbiological diagnosis can result in important benefits to patient outcomes. These include targeted and efficacious therapy, reduced risk of adverse drug reactions, and reducing the selection pressure for antimicrobial resistance in the healthcare environment. There are also potential cost savings from targeting empirical regimens with a microbiological diagnosis. Although the current cost of 16S rRNA gene sequencing remains high, its judicious use may bring clinical and economic benefits to patients and healthcare providers.

This is the largest study to date to report on the utility of 16S rRNA gene sequencing on intraoperative neurosurgical specimens and adds to smaller reports from other jurisdictions. O’Donnell *et. al.* reviewed the utility of 16S rRNA gene sequencing in a large tertiary referral adult hospital in Ireland over the course of a year (2015), including 15 neurosurgical specimens (three CSF and 12 intracranial).^8^ However, 16S rRNA gene sequencing results had a clear influence on antimicrobial choice in only three of 15 neurosurgical cases. Akram *et. al.* reported on 16S rRNA gene sequencing on 32 patient specimens from sterile sites captured over a 3 year period in New South Wales, Australia.^7^ Two of these were intracranial specimens (brain abscess; meningitis) and were reported as either negative or contaminated. In neither case did 16S rRNA gene sequencing results influence patient management. The higher rate of utility in our study may be related to the MDT protocol behind specimen selection and larger sample size.

There were some limitations to our study. First, the sample size was relatively small in spite of the 8-year study period. This is partly attributable to the protocol behind selection of specimens for 16S rRNA gene sequencing at our centre. These discussions must be timely and occur within the laboratory’s 14 day window of receiving and discarding the specimen. Therefore, specimen selection has relied heavily on the initiative and coordination of neurosurgical and infection specialists, which is also susceptible to selection bias. Some have suggested an alternative approach of routinely sending specimens for 16S rRNA gene sequencing after 72 hours if standard and enrichment cultures are negative.^10^ Trialling such a protocol would be helpful to further characterise the scenarios in which 16S rRNA gene sequencing will yield clinically-useful information. Second, it is possible that delay in transferring specimens to the reference lab from our centre resulted in degradation of genetic material and false negative reports, limiting the impact of this testing modality on decision-making. This warrants caution in relying on 16S rRNA gene sequencing to exclude infection diagnoses, whether alone or in combination with other tests.

There are also important shortcomings to current approaches to 16S rRNA gene sequencing which may restrict the generalisability of our findings. First, its specificity and sensitivity has not been decisively quantified. Since this technique amplifies any bacterial rDNA detected in the specimen, it is highly dependent on precise sampling and there remains a high risk of contamination. Second, intracranial abscesses are frequently polymicrobial and this model of 16S rRNA sequencing is not effective at detecting smaller subpopulations of bacteria. Cloned sequences may represent the dominant bacteria at the site of sampling, which may be in the abscess centre and therefore not be representative of species driving expansion of the infection at the periphery. The polymicrobial nature of brain abscesses may sway clinicians away from narrow-spectrum regimens, even when a dominant pathogen has been detected. Clinician uncertainty surrounding the accuracy of this test leads to a conservative approach to changing treatment based on the result. This may be addressed by a well-designed study to estimate the accuracy of this test.

Technical limitations may be addressed by the continued development of molecular diagnostics. Two examples of progression in technical performance are 16S-rRNA-based massive parallel sequencing (MPS)^9^ and next generation sequencing (NGS).^10^ A large Norwegian study detected 160 species from 51 intracranial specimens isolated over a 2-year period using MPS sequencing of the bacterial 16S rRNA gene, although the clinical significance of many of these species remains unclear.^9^ More recently, another group have reported 16S-rRNA NGS to identify well-described bacterial causes of intracranial infection, plus some species with less frequent association.^10^ It could also detect multiple species in polymicrobial abscess, whilst reducing the reporting of contaminants by removing low-frequency reads. However, these technologies face similar limitations to standard 16S rRNA gene sequencing of cost and accessibility. In time, molecular diagnostics may progress to combine species identification with measurable expression of antimicrobial resistance genes, giving a fuller picture more akin to culture and susceptibility testing.^11^

## Conclusion

16S rRNA gene sequencing offers an additional method to identify pathogens from culture-negative intracranial specimens and may be particularly useful in some clinical scenarios. We found it to offer moderate incremental benefit in combination with culture-based testing overall but may be better able to influence clinical decision-making with increasing experience and confidence in its diagnostic accuracy.

## References

[1] Brouwer MC, van de Beek D. Epidemiology, diagnosis, and treatment of brain abscesses. *Curr Opin Infect Dis* 2017;30:129–134. https://journals.lww.com/co-infectiousdiseases/Fulltext/2017/02000/Epidemiology,_diagnosis,_and_treatment_of_brain.18.aspx 27828809 10.1097/QCO.0000000000000334

[2] Brouwer MC, Coutinho JM, van de Beek D. Clinical characteristics and outcome of brain abscess: systematic review and meta-analysis. *Neurology* 2014; 82:806–13. Available at: http://n.neurology.org/content/82/9/806.abstract. 24477107 10.1212/WNL.0000000000000172

[3] de Louvois R, Bayston PD, Lees IK, Pople JE. The rational use of antibiotics in the treatment of brain abscess. *Br J Neurosurg* 2000; 14:525–30. Available at: 10.1080/02688690020005527. 11272029

[4] Arlotti M, Grossi P, Pea F, *et al.* Consensus document on controversial issues for the treatment of infections of the central nervous system: bacterial brain abscesses. *International Journal of Infectious Diseases* 2010; 14:S79–S92. Available at: 10.1016/j.ijid.2010.05.010. 20846891

[5] Mishra AK, Dufour H, Roche P-H, Lonjon M, Raoult D, Fournier P-E. Molecular revolution in the diagnosis of microbial brain abscesses. *Eur J Clin Microbiol Infect Dis* 2014; 33:2083–93. Available at: 10.1007/s10096-014-2166-z. 24935615

[6] Schabereiter-Gurtner C, Nehr M, Apfalter P, Makristathis A, Rotter ML, Hirschl AM. Evaluation of a protocol for molecular broad-range diagnosis of culture-negative bacterial infections in clinical routine diagnosis. *J Appl Microbiol* 2008; 104:1228–37. Available at: 10.1111/j.1365-2672.2007.03648.x 18028360

[7] Akram A, Maley M, Gosbell I, Nguyen T, Chavada R. Utility of 16S rRNA PCR performed on clinical specimens in patient management. *Int J Infect Dis* 2017;57:144–9. Available at: 10.1016/j.ijid.2017.02.006. 28216180

[8] O'Donnell S, Gaughan L, Skally M, *et al.* The potential contribution of 16S ribosomal RNA polymerase chain reaction to antimicrobial stewardship in culture-negative infection. *J Hosp Infect* 2018; 99:148–52. Available at: https://www.sciencedirect.com/science/article/pii/S0195670117304607. 28838799 10.1016/j.jhin.2017.08.016

[9] Kommedal Ø, Wilhelmsen MT, Skrede S, *et al.* Massive parallel sequencing provides new perspectives on bacterial brain abscesses. *J Clin Microbiol* 2014;52:1990–7. Available at: https://pubmed.ncbi.nlm.nih.gov/24671797. 24671797 10.1128/JCM.00346-14 PMC4042731

[10] Stebner A, Ensser A, Geißdörfer W, Bozhkov Y, Lang R. Molecular diagnosis of polymicrobial brain abscesses with 16S-rDNA-based next-generation sequencing. *Clin Microbiol Infect* 2021;27:76–82. Available at: https://pubmed.ncbi.nlm.nih.gov/24671797. 32244052 10.1016/j.cmi.2020.03.028

[11] Watts GS, Youens-Clark K, Slepian MJ, *et al.* 16S rRNA gene sequencing on a benchtop sequencer: accuracy for identification of clinically important bacteria. *J Appl Microbiol* 2017;123:1584–96. Available at: https://pubmed.ncbi.nlm.nih.gov/28940494. 28940494 10.1111/jam.13590 PMC5765505

